# Isoform specific FBXW7 mediates NOTCH1 Abruptex mutation C1133Y deregulation in oral squamous cell carcinoma

**DOI:** 10.1038/s41419-020-02873-4

**Published:** 2020-08-13

**Authors:** Yang Zheng, An Song, Chundi Wang, Wei Zhang, Dong Liang, Xu Ding, Gang Li, Hongchuang Zhang, Wei Zhang, Yifei Du, Junbo Zhou, Heming Wu, Yunong Wu, Xiaomeng Song

**Affiliations:** 1 Jiangsu Key Laboratory of Oral Diseases, Nanjing Medical University, Nanjing, Jiangsu People’s Republic of China; 2 Department of Oral and Maxillofacial Surgery, Affiliated Hospital of Stomatology, Nanjing Medical University, Nanjing, Jiangsu People’s Republic of China; 3 Department of Stomatology, Lianyungang Oriental Hospital, Lianyungang, Jiangsu People’s Republic of China; 4 Department of Stomatology, Affiliated Hospital of Xuzhou Medical University, Xuzhou, Jiangsu People’s Republic of China; 5 Department of Stomatology, Xuzhou No. 1 Peoples Hospital, Xuzhou, Jiangsu People’s Republic of China; 6 Department of Oral Pathology, Affiliated Stomatological Hospital, Nanjing Medical University, Nanjing, Jiangsu People’s Republic of China; 7 Department of Stomatology, Nanjing Integrated Traditional Chinese and Western Medicine Hospital, Nanjing, Jiangsu People’s Republic of China

**Keywords:** Oral cancer, Ubiquitylation

## Abstract

Our group previously identified that the NOTCH1 Abruptex domain contains the most mutations in Chinese OSCC patients, including a hotspot mutation (C1133Y). FBXW7 is an E3 ubiquitin ligase that regulates a network of proteins, including NOTCH1, via degradation. In this study, we first described the co-localization of isoform specific FBXW7-FBXW7β and NOTCH1^C1133Y^ mutation in the same cytoplasmic sites. Gain- and loss-of-function assays were performed to examine the tumor suppressor role of FBXW7β in the proliferation and invasion of OSCC cells. The co-expression of NOTCH1^C1133Y^ and FBXW7β significantly attenuated tumor growth. Meanwhile, FBXW7β reversed the oncogenic phenotype and the activation of the AKT/ERK/NFκB pathway induced by NOTCH1^C1133Y^ mutation. FBXW7β downregulated the stability of NOTCH1^C1133Y^ protein and promoted protein ubiquitination. This was the first time that we selected a NOTCH1 hotspot mutation detected in clinical samples and identified the function of FBXW7β that mediated NOTCH1 mutation degradation in OSCC. The newly identified interaction between FBXW7β and NOTCH1^C1133Y^ protein provides new insights into the progression of OSCC, especially regarding Abruptex domain mutations, and represents a valuable target for OSCC therapy.

## Introduction

Squamous cell carcinoma of the head and neck (HNSCC) including oral squamous cell carcinoma (OSCC) represents the sixth most common cancer worldwide, characterized by variation and a propensity for lymph node metastasis^1–3^. In China, most patients have already been in late stages when diagnosed and over 76,000 OSCC patients died each year^4^. Despite the advances in comprehensive treatment, the 5-year overall survival rate for OSCC is ~50%^2,3,5^. Therefore, it is urgent to improve our understanding of the progression of OSCC to improve the survival outcome and minimize morbidity.

The NOTCH1 protein is a transmembrane signal transducer, which is critical for developing and maintaining tissue homeostasis^6,7^. Briefly, NOTCH1 protein goes through a cleavage at the S1-site in the Golgi complex and the mature protein is expressed as a heterodimeric receptor on the cell surface. To date, 5 mammalian ligands (Jagged 1, 2 and Delta 1, 3, and 4) are widely identified^8,9^. The NOTCH1 extracellular domain (NECD) contains 36 tandem EGF like repeats that contribute to ligand binding. The ligand binding enables the NOTCH1 protein to undergo metalloprotease- and γ-secretase regulated proteolytic cleavage, which sequentially causes the delivery of the NICD (NOTCH1 intracellular domain) from the cytomembrane^10^. The NICD fraction then reaches the nucleus and interplays with the transfer factors CSL (CBF1, Suppressor of Hairless, Lag-1) family. Post-translational modification of the NOTCH1 can affect its level of activation, which subsequently influences downstream targets^11^. Except the definite function of EGF repeats 11 and 12 on the NOTCH1 receptor binding, numerous reports have verified the participation of other NOTCH1 extracellular domains in the regulation of NOTCH1 activity. Studies in Drosophila reported the involvement of the Abruptex region (EGFR repeats 24–29) of NOTCH1 in canonical NOTCH1 signaling stimulation^12,13^. A NOTCH1 Abruptex region mutation resulted in reduced activation than wild-type NOTCH1 regarding its ability to interact with ligands and caused reduced expression levels on the cell surface^14^. However, the molecular mechanisms by which how the Abruptex region mediates tumorigenesis have not been clearly elucidated.

Abnormal NOTCH1 signaling has been reported to be associated with a wide amount of tumors^15^. Previously, our group analyzed that 43% of 51 OSCC tumors collected from Chinese patients presented NOTCH1 mutations^16^. Meanwhile, the overall survival (OS) and disease-free survival (DFS) in the group of patients with NOTCH1 mutations was greatly lower than the NOTCH1 wild-type group. Among the numerous mutations, Abruptex domain (amino acids 907–1143) constituted the most mutations (13 or 31%), including a hotspot mutation-C1133Y. In T-cell acute lymphoblastic leukemia (T-ALL), NOTCH1 mutations were predominantly occurred in the PEST domain, which may prohibit proteasomal degradation and increase downstream activation^8,10^. Similarly, mutations in the extracellular domain (such as Abruptex domain) may result in attenuated NOTCH1 signaling activation. In order to gain further insight into the regulation of NOTCH1 mutation, we offered point mutation (C1133Y) in the previous study^17^. We found that this mutation prevented the canonical NOTCH1 signaling activation, producing a loss of NOTCH1 function. We discovered an oncogenic phenotype of NOTCH1^C1133Y^ mutation with the acceleration of cell proliferation and invasion in OSCC. Simultaneously, we demonstrated that the NOTCH1^C1133Y^ mutation decreased the NOTCH1 S1-cleavage in the Golgi complex. The mutation resulted in the impaired transportation of NOTCH1 from the endoplasmic reticulum (ER) to the Golgi apparatus.

FBXW7 (F-box, WD repeat domain containing 7) is the substrate recognition module that regulates a network of proteins including CYCLIN E, C-MYC, NOTCH1, and C-JUN by targeting them for degradation^18,19^. FBXW7 plays a pivotal role in cell growth suppression and tumor inhibition^20,21^. Briefly, there are three FBXW7 isoforms: FBXW7α, FBXW7β, and FBXW7γ, which are distinguished by their specific first exon^22,23^. The specific exon determines different subcellular localizations: FBXW7α is localized in the nucleus, FBXW7β localizes to the ER/Golgi within the cytosol, and FBXW7γ is predominantly nucleolar^24^. Generally, C-MYC, C-JUN, and NOTCH1 can be mediated by both FBXW7α and FBXW7β^25^. Cytoplasmic FBXW7β is also responsible for the degradation of PGC-1α and CYCLIN E and induces p53-dependent control of the cell cycle^26,27^. The FBXW7γ colocalizes with C-MYC when the proteasomes are inhibited, and regulates the accumulation of nucleolar C-MYC^28^. Phosphorylation of NICD containing a conserved phosphodegron (CPD) motif can be detected by FBXW7, thus mediated NOTCH1 ubiquitinated and proteasome degradation^29^.

In this manuscript, we first described the adverse biological roles of FBXW7β and NOTCH1^C1133Y^ mutation in OSCC cells. Overexpression of NOTCH1^C1133Y^ and FBXW7β attenuated cancer growth in vitro and in vivo. FBXW7β downregulated stability of NOTCH1^C1133Y^ protein and promoted protein ubiquitinated in the ER. This was the first time that we selected an aberrant NOTCH1 Abruptex domain mutation detected in clinical samples and identified the function of FBXW7β-mediated NOTCH1 mutation degradation in OSCC. Because the therapeutic targeting of NOTCH1 presents a dilemma to date, the successful abrogation of NOTCH1 oncogenic activity shown in this study indicates a possibility for future tumor treatment.

## Materials and methods

### Tissue samples and cell culture

All experiments were approved by the ethics committee of Nanjing Medical University (PJ-2018-042-001). In brief, we gathered 30 cancer tissues and matched adjacent normal tissues from patients with histologically diagnosed OSCC cancer from Stomatological Hospital of Jiangsu Province between 2018 and 2019. The corresponding clinicopathological data were presented in Table 1. Informed consent was signed by all of the recruited patients.

**Table 1:** Clinical features of 30 patients with OSCC.

No.	Age	Sex	Location	TNM	Differentiation
1	81	F	Gingiva	T3N0M0	Well
2	53	M	Floor of mouth	T2N0M0	Poor
3	66	M	Gingiva	T2N2bM0	Moderate
4	67	M	Floor of mouth	T2N0M0	Moderate to poor
5	62	M	Gingiva	T2N0M0	Moderate
6	61	M	Buccal	T2N2bM0	Moderate
7	62	M	Tongue	T1N0M0	Moderate to poor
8	64	M	Floor of mouth	T1N2bM0	Moderate
9	65	F	Gingiva	T2N0M0	Well
10	46	M	Tongue	T2N2bM0	Moderate to poor
11	70	M	Gingiva	T3N0M0	Moderate
12	62	M	Buccal	T3N2bM0	Moderate to poor
13	50	F	Tongue	T3N2bM0	Moderate
14	34	M	Tongue	T1N2bM0	Moderate to poor
15	51	F	Buccal	T2N1Mo	Poor
16	74	M	Buccal	T2N0M0	Moderate
17	59	M	Tongue	T2N0M0	Moderate to poor
18	57	M	Palate	T3N0M0	Well
19	65	M	Gingiva	T2N0M0	Poor
20	52	M	Palate	T2N1M0	Moderate
21	65	M	Tongue	T2N2bM0	Moderate to poor
22	67	F	Gingiva	T2N2bM0	Moderate
23	77	M	Gingiva	T3N1M0	Poor
24	54	F	Buccal	T1N2bM0	Moderate
25	66	M	Tongue	T2N2cM0	Moderate to poor
26	62	M	Oropharynx	TisN0M0	Well
27	67	F	Buccal	T1N0M0	Well
28	74	F	Gingiva	T3N2bM0	Moderate to poor
29	69	M	Gingiva	T1N2bM0	Moderate
30	63	M	Tongue	T2N2bM0	Moderate

TNM classification and tumor stage were determined by the Union for International Cancer Control (UICC).

*OSCC* oral squamous cell carcinoma, *F* female, *M* male.

Human OSCC cell lines (HN4, HN6, HN13, and CAL27) were provided as previously described^17,30^. HOK cells were purchased from the American Type Culture Collection (ATCC). All cells were incubated in the corresponding medium containing 10% fetal calf serum (FBS, HyClone, USA). Cells were cultured in a humidified atmosphere at 37 °C with 5% CO_2_. MG-132 and Cycloheximide (CHX) were bought from Selleck (Selleck Chem, Houston). Dimethyl sulfoxide (DMSO) was used for control.

### Quantitative real-time polymerase chain reaction

Cells and tissue samples were collected to extract total RNA using TRIzol (Invitrogen, Carlsbad, CA, USA) reagent and cDNA was generated using Superscript (Vazyme, Nanjing, China) according to the manufacturer’s instructions. Relative expression levels of related genes were measured by the 2^−ΔΔCT^ methods. All primers were listed as follows:

NOTCH1: F: 5′-AGCAAGTTCTGAGAGCCAGG-3′

R: 5′-TAACAGGCAGGTGATGCTGG-3′

FBXW7α: F: 5′-GAAAGCACATAGAGTGCCAAC-3′

R: 5′-TACATCTGTCCAGCCACCTAC-3′

FBXW7β: F: 5′-CCAAAAGTTGTTGGTGTTGCT-3′

R: 5′-GAAAATATGGGTTTCTACGGC-3′

FBXW7γ: F: 5′-CCAACTTTCTTTTCATCCGTCT-3′

R: 5′-CGGGAAAACCTACTCTAAACC-3′

GAPDH: F: 5′-GAAGGTGAAGGTCGGAGTC-3′

R: 5′-GAGATGGTGATGGGATTTC-3′

### Vector construction and transfection

The full-length coding region of NOTCH1 (NOTCH1^WT^), mutant NOTCH1 (NOTCH1^C1133Y^) and FBXW7β cDNA were inserted into PEGFP-N1 vectors and were generated by Generay Biotech (Shanghai, China). Cells utilized for transfection (5 × 10^5^ cells/well) were grown to ~60% confluence in recommended growth medium, and cells were starved in serum-free medium and incubated for 16 h. HN6 and CAL27 cells were transformed with the purified PEGFP-N1-NOTCH1^WT^ (referred as WT), PEGFP-N1-NOTCH1^C1133Y^ (referred as 1133Y), PEGFP-N1-FBXW7β (referred as FBXW7 β), or PEGFP-N1 (referred as NC) plasmids using Lipofectamine 2000 (Invitrogen) according to the manufacturer’s instructions. After 2 days, 200 μg/ul G418 (Gibco) was added into the medium for ~2 weeks to generate stable expressing cells.

OSCC cells were transduced using a CRISPR/Cas9 system to knock out FBXW7β or a non-targeting control in accordance to the manufacturer’s protocol. The sgRNA was selected under the assistance of the CRISPR design tool according to a standard protocol. The sgRNA oligomers were produced and cloned into the pU6gRNACas9EGFP vector. The sgRNA sequences of FBXW7β were made by Shanghai Genepharma (Shanghai, China). The sgRNA sequences were as follows: sgRNA1: 5′-CTGAGGTCCCCAAAAGTTGT-3′; and bottom strand: 5′-GAAACATTTTTAGCCATTCC-3′; sgRNA2: 5′-TGAACATGGTACAAGCCCAG-3′; and bottom strand: 5′-ACATCTGTCCAGCCACCTAC-3′; sgRNA3: 5′-TGGGAATCATTTTGGCCTCC-3′; and bottom strand: 5′-GATCAAAATCGTCACTCTCC-3′. Knockdown efficiency was determined by RT-PCR analysis after 48 h of culture.

### Western blot analysis

Western blot analysis was performed as described before^30^. The proteins were incubated with primary antibodies against FBXW7 (detecting all three isoforms, ab12292, abcam), FBXW7β (ab109617, abcam), cyclin E1 (#4129, CST), cyclin D1 (#55506, CST), CDK2 (#2546, CST), CDK4 (#12790, CST), CDK6 (#3136, CST), AKT (#4691, CST), p-AKT (#4060, CST), ERK (#4696, CST), p-ERK (#4370, CST), E-cadherin (#3195,CST), N-cadherin (ab18203), β-catenin (#8480), NF-κB p65 (#8242, CST), p-NF-κB p65 (#3033, CST), Snail (#3879, CST), Slug (#9585, CST), vimentin (#5741, CST), and β-actin (AP0733, Bioworld, China) at 4 °C overnight. The β-actin was regarded as the internal control.

### Immunofluorescence staining

Cells with stable transformed FBXW7β and NOTCH1^C1133Y^ were cultured on dishes overnight, and then fixed with 4% formaldehyde in 0.1 M phosphate buffer. Antibody against NOTCH1 was from CST (D6F11); antibody against FBXW7β was from abcam (ab109617); antibody against Calnexin was from Santa Cruz Biotechnology (SC-23954) with a dilution of 1:100 at 4 °C overnight. Then cells were washed and further incubated with FITC or Cy3-labeled goat anti-rabbit or anti-mouse IgG (Proteintech, China) at a dilution of 1:500 at room temperature for 30 min and then stained with 4′,6‐di‐amidino‐2‐phenylindole (DAPI; Sigma Chemicals). Plates were blindly examined and taken by a fluorescence microscope (DM4000B, Leica, Germany). Images were overlayed and analyzed by ImageJ software.

### Cell viability CCK‐8 assay

Stable transformed HN6 or CAL27 cells were plated at a density of 1 × 10^3^ cells/well into 96‐well plates. Cell viabilities were determined at 0, 1, 2, 3, and 4 days after cell attachment. At the end of each timing, 10 μL CCK‐8 reagent (Dojindo, Japan) was introduced to each well. Cells were then incubated for 2 h at 37 °C. The absorbance of optical density was measured at 450 nm using a Varioskan Flash Microplate Reader. Cell growth curves were plotted according to the average absorption values of each experiment. Experiments were carried out in triplicate and repeated more than twice.

### Colony formation, wound healing, and invasion assays

Following NOTCH1^C1133Y^ and FBXW7β cDNA or FBXW7β sgRNA infection, 500 cells were plated in six‐well plates. After 2 week of incubation, colonies were fixed in 5% formalin and then stained with crystal violet. Cell colony images were counted under the microscope (DM4000B, Leica, Germany) and analyzed by ImageJ. For wound healing assays, stably transformed cells were developed to ~90% confluence in six-well plates. Artificial wounds were prepared with a 200-μl sterile pipette tip across the cell surface. The cells were starved with serum-free medium and incubated to allow the cells to migrate into the open area. Images of the same area of the wound were taken at 0 and 18 h for calculating the closure of the wound. Cell invasion was measured by 24-well plates. In all, 5 × 10^4^ cells were seeded in a matrigel-coated 8-μm pore size chamber (BD Biosciences). After incubated for 24 h, cells attached to the lower layer were fixed with methanol and stained with methylene blue. The results were analyzed by counting the stained cells using microscopy (×50 magnifications) in three randomly selected fields. The experiments were repeated in triplicate and performed on two independent conditions.

### Flow cytometry cell cycle assay

In all, 1 × 10^6^ cells/well HN6 or CAL27 stable transfected cells were plated in six-well plates. Cells were then harvested and washed twice with PBS, and resuspended in 70% ice-cold ethanol for 2 days. Then cells were washed and centrifuged and resuspended with 0.5 mL propidium iodide (PI) staining buffer for 30 min in the dark at room temperature. The cell cycle profiles were assessed by FACScan cytometry at 488 nm.

### Immunoprecipitation

Cells were harvested and lysed in 600 μL of RIPA buffer (Beyotime) with protease inhibitors. Then cells were scraped up on ice and the supernatants were collected by centrifugation. The supernatants of cell lysates were interacted with indicated antibodies, GFP (ab290, abcam), NOTCH1 (D1E11, CST), or FBXW7 (ab109617, abcam) and Protein A/G PLUS-Agarose beads (Sigma-Aldrich) at 4 °C for 12 h. After immunoprecipitation, the beads were washed thoroughly with cell lysis buffer. In all, 60 μL of immunoprecipitated proteins and 1× SDS PAGE was boiled for 10 min and then the precipitated proteins were analyzed.

### Animal experiments

All animal investigations were approved by the guidelines of the Institutional Animal Care and Use Committee of Nanjing Medical University (IACUC-1601030). Generally, 36 male nude mice (5 weeks old) were bought from the Model Animal Research Institute of Nanjing University. A total of six groups were randomly assigned into six groups as followed: FBXW7β-sgRNA, FBXW7β-sgRNA/control, FBXW7β, NOTCH1^C1133Y^, FBXW7β + NOTCH1^C1133Y^, and FBXW7β/control. Stable transfected HN6 cells were centrifuged and resuspended in 50% matrigel and were subcutaneously injected into the flank of the nude mice (2 × 10^7^ cells/200 μL). Xenograft tumor size was examined by vernier caliper every 3 days, and tumor volume was measured according to the formula: volume = (length × width^2^)/2. After 21 days of injection, all nude mice were executed to assess tumor volume, weigh as well as immunostaining.

### Immunohistochemistry

In all, 10% neutral buffered formalin was used to fix the xenograft tumor specimens for 24 h followed by standard tissue processing and embedding. The tissue sections were interacted with primary antibodies against p-AKT (#4060, CST), p-ERK (#4370, CST), and p-NF-κB p65 (#3033, CST) overnight followed by conjugated secondary antibody incubation. Tissue sections were washed and counterstained with haematoxylin, dehydrated and mounted before examination utilizing a microscope (DM4000B, Leica, Germany).

### Statistical analysis

Results expressed as the mean ± SD. All images represent at least three independent experiments. Statistical significance was evaluated using Graghpad Prism 7.0. *p* < 0.05 was considered statistically significant for all tests (*p < 0.05, **p < 0.01, ***p < 0.001).

## Results

### NOTCH1^C1133Y^ mutation stimulated the cell proliferation, migration, and invasion of OSCC cells

**Fig. 1:**
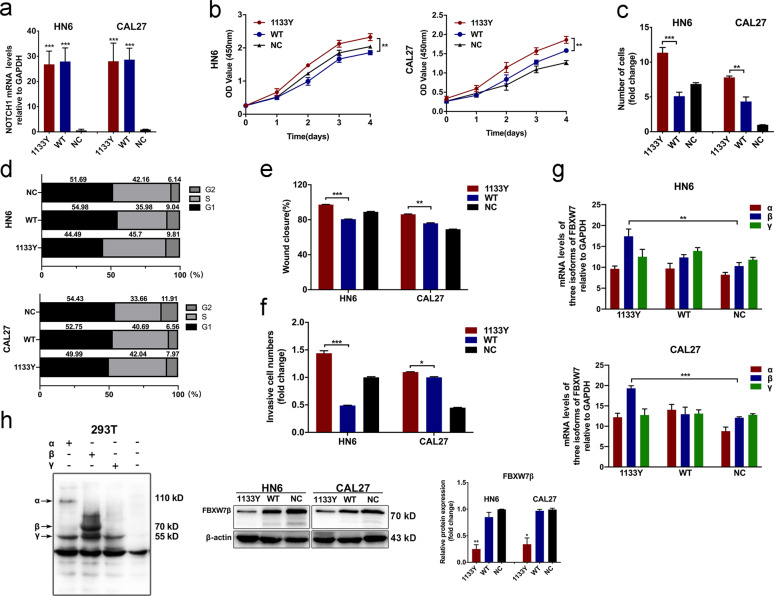
NOTCH1^C1133Y^ mutation stimulated the cell proliferation, migration, and invasion of OSCC cells. **a** Transfection efficiency of HN6 and CAL27 NOTCH1^C1133Y^ and WT cells determined by real-time qPCR. **b** Growth curves of NOTCH1^WT^-transfected cells (blue lines), NOTCH1^C1133Y^ -transfected cells (red lines) and control cells (black lines). The NOTCH1^C1133Y^ -transfected cells had higher proliferation rates compared with the NOTCH1^WT^-transfected cells. **c** Colony formation assays were performed for 2 week in six-well cell culture cluster in HN6 and CAL27 stable transfected cells. **d** NOTCH1^C1133Y^- transfected cells presented a significantly lower percentage of G1 phase and higher ratio of S phase than NOTCH1^WT^ cells. **e**, **f** Wound healing and Transwell assays were employed to analyze the cell migration and invasion ability. NOTCH1^C1133Y^-transfected cells exhibited higher metastatic ability in HN6 and CAL27 cell lines compared with NOTCH1^WT^-transfected cells. **g** mRNA expression levels of three isoforms of FBXW7 (relative to GAPDH) after transfection of NOTCH1^C1133Y^ or NOTCH1^WT^ in HN6 and CAL27 cells. FBXW7β mRNA was greatly reduced in NOTCH1^C1133Y^ transfected cells compared to FBXW7α or FBXW7γ mRNA levels. **h** Left panel, the band of FBXW7 were detected by western blot after transfection of three individual isoform plasmids. Right panel, the alteration of FBXW7β protein levels in NOTCH1^C1133Y^ overexpressed cells. Data are mean ± SD from three independent experiments. *p < 0.05, **p < 0.01, ***p < 0.001.

In order to determine the function of NOTCH1^C1133Y^ mutation in tumor progression and metastasis, we first performed gain-of-function assays in HN6 and CAL27 cell lines that express a low range of endogenous NOTCH1 compared with other OSCC cell lines (data not shown). After a 2-week selection, the efficiency of infection was confirmed by qRT- PCR. Marked increase of NOTCH1 expression level was observed in Fig. 1a. In CCK-8 assays, NOTCH1^C1133Y^ overexpression significantly accelerates the proliferation of HN6-NOTCH1^C1133Y^ and CAL27-NOTCH1^C1133Y^ transfected cells compared with the controls that transfected with NOTCH1^WT^ plasmids (Fig. 1b). Colony formation and Flow cytometry cell cycle assays verified the above results (Fig. 1c, d). We then investigated the role of NOTCH1^C1133Y^ mutation on the ability of migration and invasion in OSCC cells. In wound healing studies, cell migration rate was markedly increased in the NOTCH1^C1133Y^ transfected cells compared with wide-type NOTCH1-transfected cells (Fig. 1e). As shown in Fig. 1f, we discovered that the stable expressed NOTCH1^C1133Y^ mutant cells passed through the matrix quicker than the wide-type group. These results showed that NOTCH1^C1133Y^ mutation promoted cell proliferation, migration, and invasion in OSCC cells. We then tested the mRNA levels of three FBXW7 isoforms (Fig. 1g). Levels of FBXW7β mRNA was greatly reduced in NOTCH1^C1133Y^ transfected cells compared to FBXW7α or FBXW7γ mRNA levels, although FBXW7α presented relatively high endogenous level in OSCC cells. Moreover, protein expression levels were demonstrated in the manuscript. To confirm the band position of three isoforms of FBXW7, we first separately transfected three isoform plasmids into 293T cells. Three individual bands against FBXW7 (ab12292, abcam) were shown in Fig. 1h. We then discovered the alteration of FBXW7β protein levels in NOTCH1^C1133Y^ overexpressed cells using specific antibody which binds to FBXW7β (ab109617, abcam, Fig. 1h left). Results showed that FBXW7β levels were considerably reduced compared with the wild-type or negative control (Fig. 1h right).

### FBXW7β interacted with NOTCH1^C1133Y^ in the endoplasmic reticulum

**Fig. 2:**
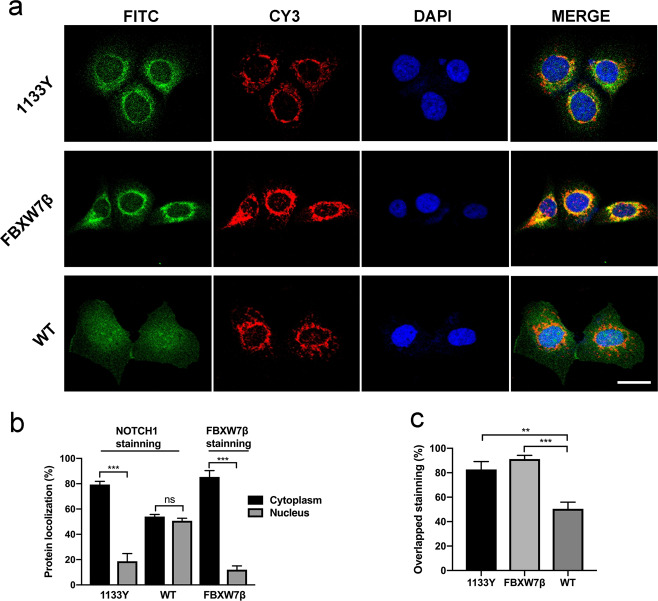
FBXW7β interacted with NOTCH1^C1133Y^ in the endoplasmic reticulum. **a** The subcellular location of NOTCH1 receptors in CAL27 cells was assessed by immunofluorescence. The NOTCH1-FITC staining revealed that NOTCH1 protein in C1133Y-mutated cells was only localized in the cytoplasm. Costaining of NOTCH1 with ER-marker Calnexin showed strong overlapped staining in NOTCH1^C1133Y^-transfected cells. FBXW7β-EGFP staining was present in the microsomal fraction together with the ER-resident protein Calnexin. Scale bar, 20 μm. **b** The localization of NOTCH1 or FBXW7β in cytoplasm or nucleus was assessed in 100 cells, and the percent of cells was shown. The data indicated that 79.3% of NOTCH1^C1133Y^-transfected cells and 85.3% of FBXW7β-transfected cells exhibited cytoplasmic expression, but that only 54% of NOTCH1^WT^-transfected cells exhibited cell cytoplasmic expression. **c** Overlapped staining of NOTCH1 or FBXW7β with ER-marker Calnexin was counted in NOTCH1^C1133Y^ and NOTCH1^WT^ or FBXW7β transfected cells. In all, 82.8% of NOTCH1^C1133Y^-transfected cells and 91.2% of FBXW7β-transfected cells showed overlap between FITC (NOTCH1 or FBXW7β staining) and CY3 (Calnexin staining), while 50.5% of NOTCH1^WT^-transfected cells showed overlapped staining between NOTCH1 and Calnexin. Data are mean ± SD. Percentages of localization were calculated from three independent experiments. **p < 0.01, ***p < 0.001.

To determine the mechanism through which FBXW7β regulates NOTCH1^C1133Y^ in OSCC cells, we doubted the direct interplay between the FBXW7β and NOTCH1^C1133Y^ protein. Although FBXW7 has been described to be involved in NOTCH1 protein degradation, no literatures have reported the interplay between FBXW7β and mutant NOTCH1 in the Abruptex domain yet. We have previously found that the NOTCH1^C1133Y^ mutation led to the retention of NOTCH1 protein in the endoplasmic reticulum and reduced the transport of full-length NOTCH1 protein from endoplasmic reticulum to the Golgi apparatus^17^. Matsumoto et al.^31^^,^^32^ reported that FBXW7β includes a supposed transmembrane domain and suggested that it substantially penetrates the ER membrane. We further examined the co-localization of NOTCH1^C1133Y^ or FBXW7β with NOTCH1 (FITC) or FBXW7β (FITC) and Calnexin (CY3) antibodies by immunofluorescence staining for intracellular expression and location. Subcellular localization showed that both NOTCH1^C1133Y^ and FBXW7β were cytoplasmic, as expected (Fig. 2a). Immunofluorescence analysis demonstrated that FBXW7β was discovered in the ER-resident protein Calnexin as reported earlier in other cell types. Transfected NOTCH1^C1133Y^ cells showed a mesh-like pattern that colocalized with Calnexin, suggestive of co-expression of FBXW7β and NOTCH1^C1133Y^ to the ER (Fig. 2b, c). Meanwhile, NOTCH1^WT^ cells did not present an evident ER localization.

### FBXW7β expression in specimens and OSCC cell lines

**Fig. 3:**
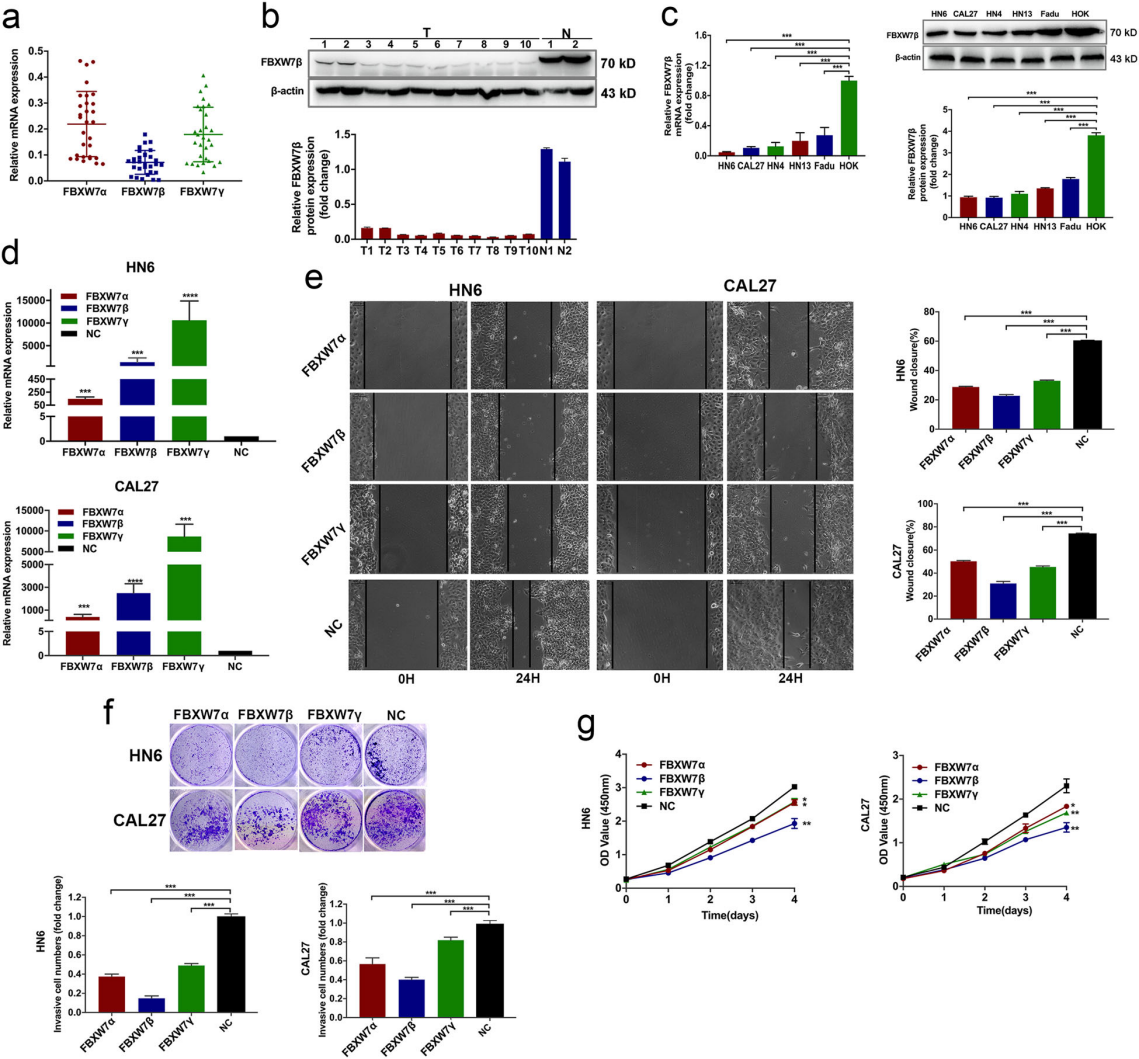
FBXW7β expression in OSCC specimens and cell lines. **a** Fold change of FBXW7β mRNA levels in 30 paired OSCC tissues. **b** Fold change of FBXW7β protein expression levels in eight OSCC tissues and two nontumor tissues. **c** Relative FBXW7β mRNA and protein levels in five OSCC cell lines and one normal oral epithelial cell line (HOK). **d** qRT-PCR detection after transfection of PEGFP-N1-FBXW7α or PEGFP-N1-FBXW7β or PEGFP-N1-FBXW7γ and empty vector in HN6 and CAL27 cells. **e**–**g** Wound healing (**e**), Transwell (**f**), and CCK-8 (**g**) assays were employed to analyze the cell migration, invasion, and proliferation ability. Compared with other two subtypes, overexpression of FBXW7β significantly reduced cell growth, migration, and invasion. Data are mean ± SD from three independent experiments. *p < 0.05, **p < 0.01, ***p < 0.001.

To discover the role of FBXW7β in OSCC tissues, we first detected the expression levels of three FBXW7 isoforms in 40 OSCC specimens and the correspondent normal tissues by quantitative real-time polymerase chain reaction (qRT-PCR) and immunoblotting assay. The qRT-PCR results demonstrated that the alteration of FBXW7β mRNA levels in tumor tissues was greatly lower than that in the correspondent normal tissues (Fig. 3a). In addition, the immunoblotting assays showed that FBXW7β protein levels were reduced in the OSCC tissues **(**Fig. 3b**)**, which was accordant with the qRT-PCR results. We then assessed the mRNA and protein levels of FBXW7 in OSCC cell lines. Results indicated that all OSCC cell lines expressed lower levels of FBXW7 than HOK cells (Fig. 3c), indicating its negative role in tumor progression.

**Fig. 4:**
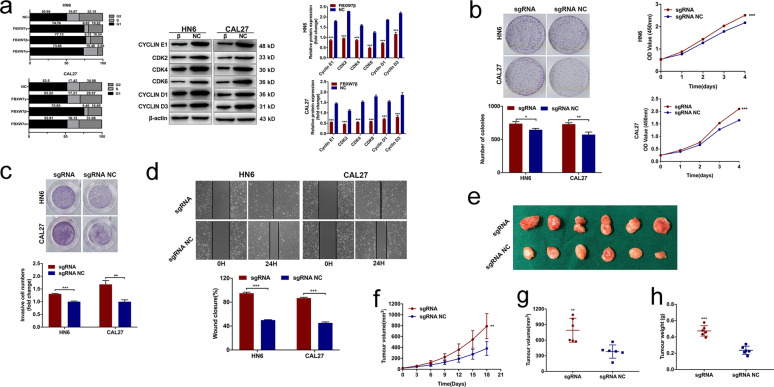
FBXW7β resulted in cell growth inhibition in OSCC cell lines. **a** Flow cytometry analysis showed cell cycle arrest due to FBXW7β transfection (left panel). Proteins related to cell cycle progression were demonstrated (middle and right panel). FBXW7β overexpression resulted in the decreased expression levels of cell cycle related proteins. **b** FBXW7β-sgRNA dramatically increased the number of colonies (left panel) and cell growth (right panel). **c**, **d** Depleting FBXW7β potently promoted cell migration (**c**) and invasion (**d**). Data are mean ± SD from three independent experiments. **e** The tumors dissected from mice (*n* = 6 for each group) were presented. **f**–**h** Evaluation on tumor incidence (**f**), weight (**g**), and size (**h**). All the results were shown as mean ± SD. *p < 0.05, **p < 0.01, ***p < 0.001.

To further investigate the role of three isoforms of FBXW7 in OSCC cells, we stably transfected PEGFP-N1-FBXW7α or PEGFP-N1-FBXW7β or PEGFP-N1-FBXW7γ and empty vector in HN6 and CAL27 cells. qRT-PCR results showed efficient transfection in both cells (Fig. 3d). Cell proliferative, migratory, and invasive abilities were assessed in Fig. 3e–g. Compared with other two subtypes, overexpression of FBXW7β significantly reduced cell growth and invasion. We then analyzed CYCLIN E1 (proved to be the specific substrate of FBXW7β) and related cell cycle proteins. Flow cytometry analysis showed cell cycle arrest due to FBXW7β transfection (Fig. 4a). Consistently, FBXW7β downregulated E‐type cyclins as well as cyclin-dependent kinases (Fig. 4a).

To investigate the biologic properties affected by FBXW7β expression, we knocked down FBXW7β in HN6 and CAL27 cells through CRISPR/Cas9 system. Three different sgRNA constructs were verified using qRT-PCR and the most effective sequence was provided in this study (Fig. S1). FBXW7β suppression dramatically increased cell growth and induced the number of colonies (Fig. 4b). Moreover, depleting FBXW7β potently promoted cell migration and invasion. (Fig. 4c, d). We then observed the tumor formation in a mouse xenograft assay. FBXW7β-sgRNA expression significantly induced tumor growth compared with control group (Fig. 4e–h).

### FBXW7β regulated NOTCH1^C1133Y^-induced oncogenic phenotype alteration

**Fig. 5:**
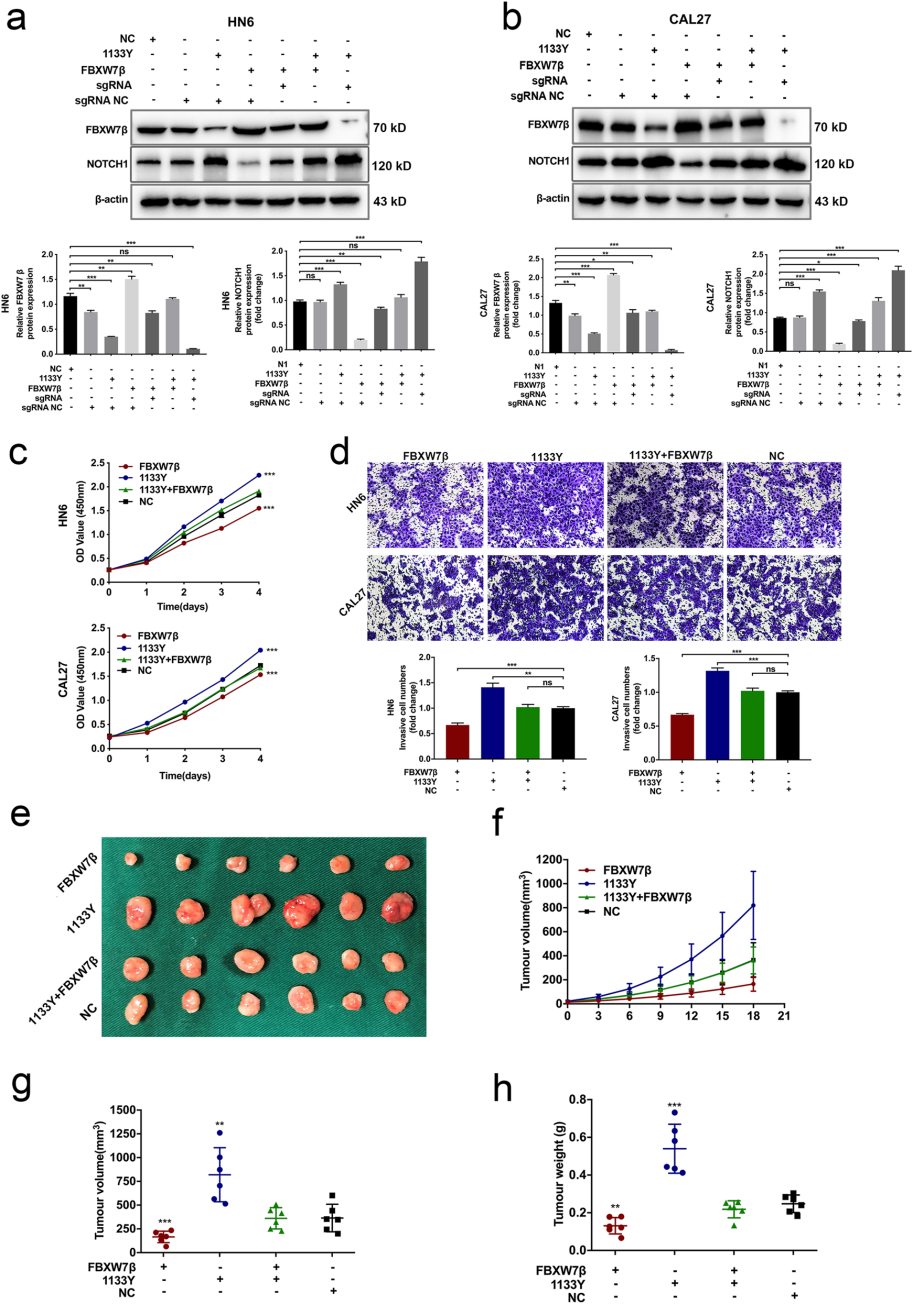
FBXW7β regulated NOTCH1^C1133Y^-induced oncogenic phenotype. **a**, **b** Western blotting was used to detect the expression levels of NOTCH1 and FBXW7β. The overexpression of NOTCH1^C1133Y^ decreased FBXW7β expression, whereas the upregulation of FBXW7β attenuated the loss of FBXW7β expression in NOTCH1^C1133Y^ overexpressed HN6 and CAL27 cells. **c** CCK-8 assay showed that upregulation of NOTCH1^C1133Y^ dramatically increased the cell proliferation ability in HN6 and CAL27 cells. **d** Transwell assay showed that the upregulation of FBXW7β significantly reduced the cell invasion in NOTCH1^C1133Y^ transfected cells. Data are mean ± SD from three independent experiments. **e** The tumors dissected from mice were presented (*n* = 6 for each group). From top to bottom, each line of tumors represented: FBXW7β, NOTCH1^C1133Y^, FBXW7β+ NOTCH1^C1133Y^, and NC. **f**–**h** Evaluation on tumor incidence, weight, and size. All the results were shown as mean ± SD. *p < 0.05, **p < 0.01, ***p < 0.001.

To validate that FBXW7β could mediate NOTCH1^C1133Y^-induced cell proliferation and invasion, we first increased or decreased the level of FBXW7β in NOTCH1^C1133Y^overexpressing HN6 and CAL27 cells. Immunoblotting analysis was used to detect the FBXW7β and NOTCH1 expression levels. As illustrated in Fig. 5a, b, the western blotting demonstrated that NOTCH1^C1133Y^ overexpression lowered FBXW7β expression. Meanwhile, the upregulation of FBXW7β prevented the loss of FBXW7β expression in NOTCH1^C1133Y^ overexpressed OSCC cells. We then decreased the expression of FBXW7β in NOTCH1^C1133Y^-overexpressing OSCC cells. Knockdown of FBXW7β expression further decreased FBXW7β expression downregulated by NOTCH1^C1133Y^ in OSCC cells. The upregulation of NOTCH1^C1133Y^ dramatically increased the cell proliferation and invasion abilities, whereas the upregulation of FBXW7β turned over the oncogenic phenotype induced by NOTCH1^C1133Y^. Simultaneously, the depletion of FBXW7β significantly enhanced NOTCH1^C1133Y^-mediated cell proliferation and invasion (Fig. 5c, d).

We then performed xenograft tumorigenesis experiments by inoculating HN6 cells expressing NOTCH1^C1133Y^, FBXW7β, or co-transfection in the flanks of nude mice and used mock-vehicle HN6 cells as the control (Fig. 5e). Proliferative curve, volumes, and weight of tumors were presented in Fig. 5f–h. Mice implanted cells expressing FBXW7β developed the smallest tumors, whereas NOTCH1^C1133Y^ significantly promoted tumorigenesis in vivo. Co-transfection of NOTCH1^C1133Y^ and FBXW7β reduced the tumorigenic ability acquired from NOTCH1^C1133Y^ in nude mice, implicating the important functional role of FBXW7β in OSCC.

### FBXW7β is critical for the activation of AKT/ERK/NFκB pathway prompted by NOTCH1^C1133Y^ mutation in OSCC cells

**Fig. 6:**
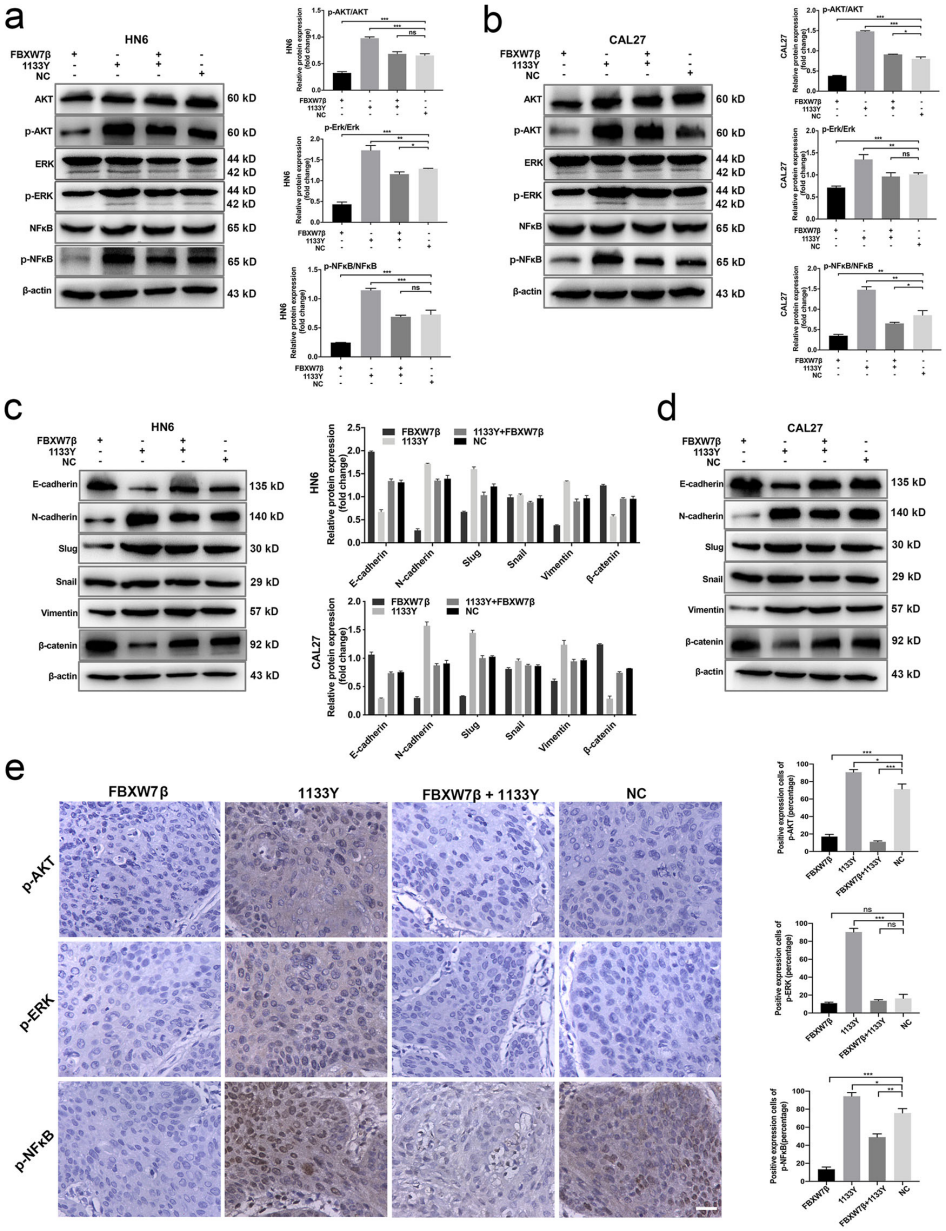
FBXW7β is critical for the activation of AKT/ERK/NFκB pathway prompted by NOTCH1^C1133Y^ mutation in OSCC cells. **a**, **b** AKT/ERK/NFκB signaling activities were evaluated by western blot analysis in HN6 (**a**) and CAL27 cells (**b**). The gray values of images demonstrated that FBXW7β transfection decreased phosphorylation of AKT/ERK/NFκB and reversed the NOTCH1^C1133Y^-induced activation of AKT/ERK/NFκB phosphorylation. **c**, **d** Overexpression of FBXW7β reversed the increased protein expression levels of EMT markers and prevented the decrease in E-cadherin and β-Catenin caused by NOTCH1^C1133Y^ in HN6 (**c**) and CAL27 (**d**) cells. **e** The expression of p-AKT, p-ERK, and p-NFκB in xenografted mice was ascertained using IHC assay on tumor sections. The percentages of positive cells were acquired from three separate images and the qualification was presented. Scale bar, 20 μm. All the results were shown as mean ± SD. *p < 0.05, **p < 0.01, ***p < 0.001.

AKT/ERK/NFκB signaling pathway contributes to cell fate decisions and promotes cell proliferation and invasion in various cancers, such as breast and colon cancers^33,34^. Meanwhile, EMT process is modulated through multiple signaling pathways including the AKT/ERK/NFκB pathway^35^. Our previous results revealed that NOTCH1^C1133Y^ could activate AKT and PI3K protein expression levels and induced EMT in OSCC cell lines. Here, we measured the levels of AKT, ERK, and NFκB in NOTCH1^C1133Y^-overexpressing HN6 and CAL27 cells with or without transfection of FBXW7β. We found that NOTCH1^C1133Y^ promoted the production of p-AKT, p-ERK, and p-NFκB compared with the changing levels of the total proteins (Fig. 6a, b). Meanwhile, FBXW7β transfection decreased the phosphorylation of AKT/ERK/NFκB and reversed the NOTCH1^C1133Y^-induced activation of AKT/ERK/NFκB phosphorylation (Fig. 6a, b). Overexpression of FBXW7β also reversed the increased expression levels of N-cadherin, Vimentin, and Snail, and prevented the decrease in E-cadherin and β-catenin caused by NOTCH1^C1133Y^ (Fig. 6c, d).

The expression levels of p-AKT, p-ERK, and p-NFκB in HN6-transplanted mice was determined using an IHC assay on tumor tissue sections. Immunohistochemical analysis revealed that compared with the NOTCH1^C1133Y^ group, the levels of phosphorylated AKT, ERK, and NFκB significantly decreased due to the co-transfection of FBXW7β and NOTCH1^C1133Y^ (Fig. 6e).

These data suggest that FBXW7β suppresses the cancer cell properties and EMT induced by NOTCH1^C1133Y^ through its effects on the AKT/ERK/NFκB signaling pathway.

### FBXW7β ubiquitination mediated NOTCH1^C1133Y^ deregulation

**Fig. 7:**
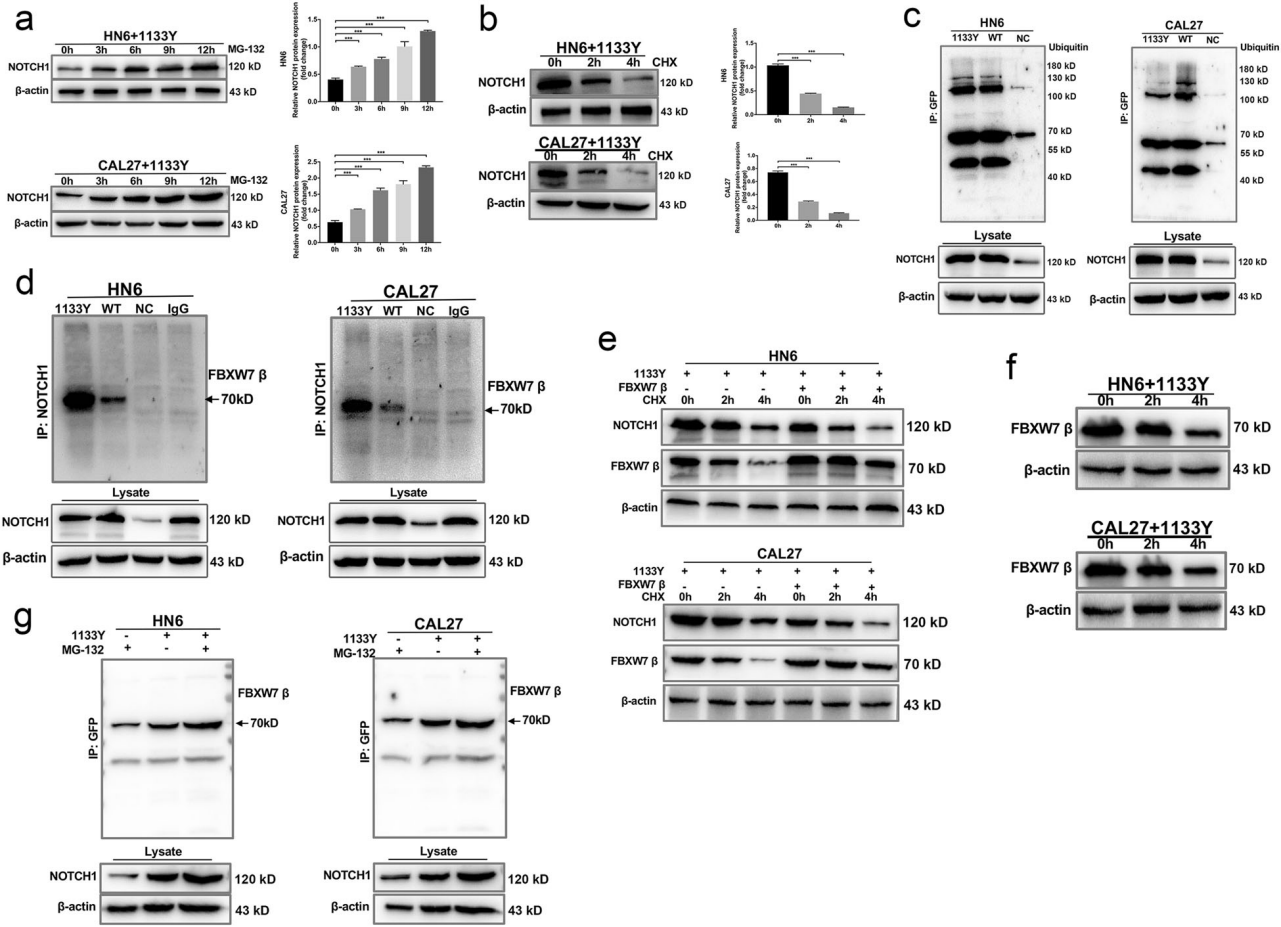
FBXW7β ubiquitination mediated NOTCH1^C1133Y^ deregulation. **a** HN6 and CAL27 NOTCH1^C1133Y^-transfected cells were treated with MG132 (10 μM) for the indicated times, and then the levels of NOTCH1 were detected. **b** The cells were subjected to cycloheximide (CHX) (20 μM) exposure at the indicated times, and the protein expression levels of NOTCH1 were verified. **c** Co-IP between NOTCH1^C1133Y^ and ubiquitin in HN6 and CAL27 cells. **d** Co-IP experiment showed that NOTCH1^C1133Y^ could be co-precipitated together with FBXW7β. HN6 or CAL27 cells were transfected with NOTCH1^C1133Y^, NOTCH1^WT^, or NC plasmids and precipitated with NOTCH1 antibody. IgG group presented the lysates of cells transfected with NOTCH1^C1133Y^ were precipitated with igG, which represented the negative control. **e** Detection of the effects of NOTCH1^C1133Y^ on FBXW7β expression, either with or without CHX (20 μM) in HN6 and CAL27 cells. **f** Ectopic dose-dependent effect of NOTCH1^C1133Y^ overexpression caused a significant reduction of endogenous FBXW7β proteins. **g** Co-IP experiment showed MG-132 promoted the binding level of NOTCH1 and FBXW7β. The cells in NOTCH1^C1133Y^ groups were treated with or without proteasomal inhibitor MG132 (10 μM). Cell lysates were prepared and subjected to immunoprecipitation with anti-GFP antibody. The level of FBXW7β was detected by western blotting analysis. Data are mean ± SD from three independent experiments. ***p < 0.001.

Considering that NOTCH1 is a novel substrate of FBXW7, we investigated whether NOTCH1^C1133Y^ protein was under the control of the FBXW7β ubiquitin-proteasome system (UPS) in the ER, which is the main process for the degradation of cytoplasmic proteins^19,36^. NOTCH1^C1133Y^ overexpression demonstrated that NOTCH1^C1133Y^ accumulation was related to the amount and the length of incubation to the proteasome inhibitor MG-132 (Fig. 7a). Cycloheximide (CHX) experiments were then carried out in NOTCH1^C1133Y^ overexpressing cells. The NOTCH1 protein level was increased in response to proteasomal inhibition (Fig. 7b). To determine whether the degradation and ubiquitination of NOTCH1^C1133Y^ proteins occurred in OSCC, we investigated the interplay between NOTCH1^C1133Y^ and ubiquitin. The Co-IP analysis presented that the NOTCH1^C1133Y^-EGFP protein and ubiquitin were detected in the immunoprecipitate experiment (Fig. 7c). This result indicated that ubiquitin-proteasome system also participated in the NOTCH1^C1133Y^ protein degradation.

We then doubted whether the NOTCH1^C1133Y^ protein levels would be deregulated by FBXW7β. Co-IP experiment showed that NOTCH1^C1133Y^ could be co-precipitated together with FBXW7β (Fig. 7d). To verify whether FBXW7β took part in the degradation of NOTCH1^C1133Y^ protein, we transduced the NOTCH1^C1133Y^ plasmids into HN6 and CAL27 cells. The effects of NOTCH1^C1133Y^ on FBXW7β expression were detected with or without CHX incubation (Fig. 7e). The degradation dynamics assay revealed that the half-life of NOTCH1^C1133Y^ was greatly shortened in the FBXW7-overexpressing cells compared with that in the control cells. We then explored the effect of FBXW7β in the procedure of NOTCH1^C1133Y^ degradation. Western blot analysis revealed that the excessive dose-dependent influence of NOTCH1^C1133Y^ overexpression resulted in a marked reduction of endogenous FBXW7β protein (Fig. 7f). Meanwhile, NOTCH1^WT^ overexpression caused a significant FBXW7α protein level reduction (data not shown). Finally, we introduced MG132 to HN6 and CAL27 cells transduced with NOTCH1^C1133Y^ and lysates were incubated with the GFP antibody for immunoprecipitation (Fig. 7g). We discovered that MG-132 promoted the binding level of NOTCH1 and FBXW7β. To conclude, these results suggested that FBXW7β mediated NOTCH1^C1133Y^ expression by regulating its ubiquitination.

## Discussion

NOTCH1 is an extremely conservative transmembrane receptor that transports intercellular signals to regulate cell fate^6^. Recently, the non-canonical stimulation of NOTCH1 has also been correlated with tumorigenic events in various cancers. Previous studies have shown that the Abruptex domain of NOTCH1 is necessary to mediate the functions of ligands that result in the suppression of NOTCH1 activity^12,13,37^. In OSCC, a comprehensive analysis of genomic alterations was constructed, and a vast number of NOTCH1 mutations have been identified. Remarkably, compared with the reported incidence of 14 and 15% of NOTCH1 mutations among Caucasian patients^38,39^, more than a half of Chinese patients harbor missense NOTCH1 mutations. Moreover, patients with mutations show a markedly worse OS and DFS than those with the wild-type form, emphasizing the pivotal role of NOTCH1 mutations in Chinese patients suffered from OSCC.

We have previously confirmed the subcellular localization of the NOTCH1 hotspot mutation - NOTCH1^C1133Y^ and observed the tumorigenic phenotype in OSCC cells. We observed that compared with wide-type NOTCH1, NOTCH1^C1133Y^ proteins were generally accumulated in the endoplasmic reticulum. However, the mechanism on the accumulation of this NOTCH1 mutation is still unknown. Post-translational modification of the NOTCH1 proteins can influence their level of activation, which subsequently affects downstream targets. One of the important processes is glycosylation^40^. It has been proved that some of the EGF repeats (including Abruptex domain) existed in NOTCH1 can be modified by two particular subtypes of protein glycosylation: O-fucose and O-glucose in the ER^12^. Presence of the O-fucose residues in the EGF repeats initiated by the Fringe enzymes prevents the interaction of NOTCH1 with Jagged ligands. Removal of single O-fucose sites on Mouse NOTCH1 Abruptex domain could alter NOTCH1 activation ability in cell-based manners. In addition, loss of O-fucosyltransferase 1 (Pofut1), which mediated the O-fucose, resulted in a NOTCH1 loss-of-function phenotype due to varied temperature exposure. Because O-fucose modification is essential for NOTCH1 maturation and activation, the individual mutation in the Abruptex domain may render a NOTCH1 comformational alteration, thus results in its misfolding and accumulation in the endoplasmic reticulum. Interestingly, although the Abruptex region has several glycosylated sites, the region where C1133Y presents (EGF 29) does not have similar consensus sequence^12,13,37^. Therefore, the exact mechanistic details responsible for the retention of this NOTCH1 Abruptex mutation remain to be clarified by future studies.

Multiple structural researches have offered insights into the interplay between the NOTCH1 phosphorylation and FBXW7 binding. NOTCH1 contains a conservative Cdc4 phosphodegron (CPD) motif that interacts with FBXW7 phosphate-binding pockets^41^. Phosphorylated forms of NICD have been identified within the nucleus and have been associated with CSL members and signaling activity. Glycogen synthase kinase 3β (GSK3β) can phosphorylate the PEST domain (around threonine 2512, T2512) of NICD thus results in the NICD degradation^19,42^. In this study, although we did not test the level of phosphorylation on NOTCH1^C1133Y^, we observed the direct interaction between NOTCH1^C1133Y^ and FBXW7β. Further experiments still needed to be conducted in regard to the activation of GSK3β and the phosphorylation status of NOTCH1. The aberrant activation of the AKT/ERK/NFκB signaling pathway is associated with a variety of pathological alterations^43,44^. It has been hypothesized that by connecting FBXW7-mediated degradation with GSK3β activity, AKT/ERK signaling can synchronously stabilize several downstream proteins. Likewise, AKT/ERK may also stabilize NOTCH1^C1133Y^ protein by competing with GSK3β and downregulate the affinity between NOTCH1^C1133Y^ and FBXW7β. In this study, we tested the expression levels of AKT, ERK, and NFκB in NOTCH1^C1133Y^ overexpressed cells with or without transfection of FBXW7β. We found that NOTCH1^C1133Y^ elevated the protein levels of p-AKT, p-ERK, and p-NFκB compared with the control group. On the contrary, FBXW7β transfection decreased phosphorylation of AKT, ERK, and NFκB and reversed the NOTCH1^C1133Y^-induced activation of AKT, ERK, and NFκB phosphorylation. These results demonstrated that AKT/ERK/NFκB signaling pathway was activated in the presence of NOTCH1^C1133Y^, which could be eventually inhibited by FBXW7β.

We then investigated the mechanism by which FBXW7β mediates the NOTCH1^C1133Y^ degradation. Ubiquitin-proteasome-mediated degradation of NOTCH1 is a pivotal mechanism for NOTCH1 degradation in cancer cells. However, no literatures have verified the degradation of NOTCH1 mutations in OSCC. Our study first confirmed that NOTCH1^C1133Y^ can also be degraded by a ubiquitin system in OSCC cells. Meanwhile, we confirmed that FBXW7β participated in the degradation of NOTCH1^C1133Y^. FBXW7β overexpression promoted NOTCH1^C1133Y^ ubiquitination and degradation. We then found that FBXW7β can decrease the half-life of NOTCH1^C1133Y^ in a dose-dependent effect. Increased FBXW7β significantly induced the levels of NOTCH1^C1133Y^ ubiquitination. We also used the Yeast two-hybrid assay to verify the physical interaction between NOTCH1^C1133Y^ and Fbxw7β (data not shown). Surprisingly, we did not discover a direct activation through the Yeast two-hybrid system. The exceeded length of NOTCH1^C1133Y^ full-length protein may lower the possibility of interaction between the two proteins in vitro. Ubiquitin-proteasome-mediated degradation is a transient process. The Yeast two-hybrid system may not reflect the instantaneous binding and the degradation between the two proteins. It is also possible that other proteins might be involved in this rapid degradation.

In summary, this was the first time that we selected a NOTCH1 Abruptex mutation (NOTCH1^C1133Y^) detected in clinical samples and identified the tumorigenic property in OSCC cells. We demonstrated that FBXW7β reversed the oncogenic phenotype and activation of AKT/ERK/NFκB pathway induced by NOTCH1^C1133Y^ and regulated NOTCH1^C1133Y^ ubiquitination and degradation. Because comprehensive mutations of NOTCH1 gene have been detected in Chinese patients, the newly identified interaction between FBXW7β and NOTCH1^C1133Y^ may develop a novel view into the degradation of NOTCH1 in OSCC cells especially with Abruptex domain mutations and provided a potential target for OSCC therapy.

## Supplementary information

Figure S1

Supplementary materials-Figure legend
