# Supplementary figures and images for: Isoform specific FBXW7 mediates NOTCH1 Abruptex mutation C1133Y deregulation in oral squamous cell carcinoma
Source: Cell Death Dis. 2020 Aug 13;11(8):615. doi: 10.1038/s41419-020-02873-4 (PMC7426429; doi:10.1038/s41419-020-02873-4)

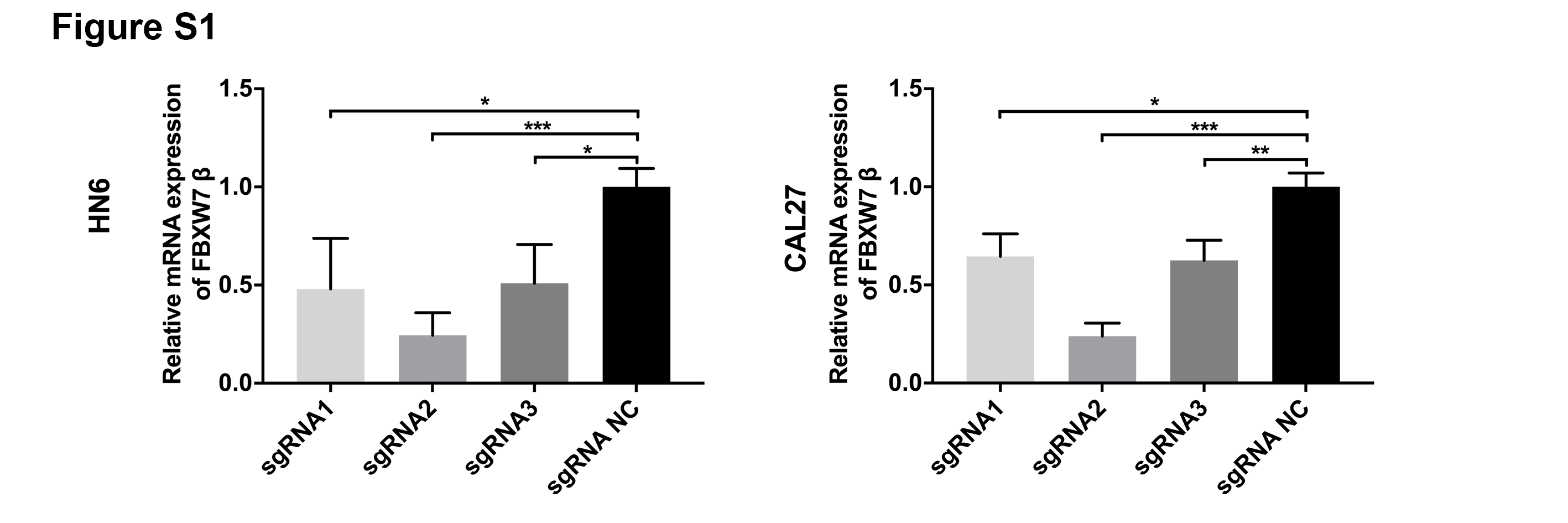

Supplement: Supplementary file 1 — Figure S1 [file 41419_2020_2873_MOESM1_ESM.tif]
