# Supplementary material for: Isoform specific FBXW7 mediates NOTCH1 Abruptex mutation C1133Y deregulation in oral squamous cell carcinoma
Source: Cell Death Dis. 2020 Aug 13;11(8):615. doi: 10.1038/s41419-020-02873-4 (PMC7426429; doi:10.1038/s41419-020-02873-4)
Supplement: Supplementary file 2 — Supplementary materials-Figure legend [file 41419_2020_2873_MOESM2_ESM.docx]

**Supplementary figure legend**

**Figure S1.** CRISPR/Cas9 system was used to knock out FBXW7β in HN6 (A) and CAL27 (B) cells. Transfection efficiency of three different sgRNA constructs were verified using qRT-PCR. SgRNA2 was utilized in the study.
